# Editorial: Health Technologies: a challenge to tackle in NETWORK

**Published:** 2020-02-20

**Authors:** M Illario, V De Luca, UM Bracale, G Bracale

**Affiliations:** 1 Department of Public Health, University Federico II of Naples, Naples, Italy; 2 Health Innovation Division, General Directorate for Health, Campania Region, Naples, Italy; 3 Research and Development Unit, Federico II University Hospital, Naples, Italy; 4 Vascular Surgery Unit, Department of Public Health, University Federico II of Naples, Naples, Italy; 5 The Mediterranean Federation for Advancing Vascular Surgery, Naples, Italy

## I. EDITORIAL

Health and social challenges require the adoption of new approaches to prevention, diagnostics and care, which pose important sustainability and equity issues to the different healthcare systems of Italian regions. It is therefore essential to set up sustainable models, as part of an overall health innovation process, where ongoing structural reforms are able to increase the effectiveness and resilience of health systems. The participation of citizens, patients, formal and informal caregivers in the planning, set-up and evaluation of these new solutions is pivotal to overcome those current approaches which are no longer fulfilling the provision of integrated social and health services.

Digital transformation of health and care provides tools capable to support the modernization of social and health systems, and their adaptation to challenges such as the progressive population ageing, especially in a framework of shared resources and skills, that bring the citizen at the center of the healthcare politics, addressing health needs at individual and community level.

The adoption of advanced technologies for diagnostics and therapy, and the digitization of services and care, represent an opportunity to be seized to set a virtuous circuit connecting needs, innovation and investments, through the adoption of transparent procedures.

The collaborative approach to the provision of health services through network models allows the multidisciplinary management of the innovative tools that are progressively adopted, while supporting operators training, citizen empowerment and outcomes monitoring, also through rationalization and centralized management of financial resources. With these premises, on 1st October 2018 MeFAVS, the Mediterranean Federation for Advancing of Vascular Surgery was founded, willing to connect University Professors, heads of Vascular Departments and consultant surgeons for ongoing scientific, educational and clinical cooperation amongst the Mediterranean basin countries, such as Italy, France, Spain, Portugal, Greece, Morocco, Algeria, Tunisia, Egypt, Lebanon, Emirates, Albania, Croatia and Turkey, among others. Its activities, some currently and actively ongoing, have been a series of verbal information exchanges, meetings and surveys based on common topics of vascular pathology, epidemiology, new treatments and materials for Vascular Surgery. This project was born in collaboration with the “Federico II” University of Naples and the Campania Region which, according to the European Community directives and regulations, aimed to include MeFAVS in the Pro.M.I.S. (“Progetto Mattone Internazionale” Italian Ministry of Health Programme for Internationalization of Regional Health Systems) linked to the Horizon 2020 cycle to gain access to European Community funds managed by the Regional Governance.

During a two-days meeting held in Pozzuoli (Naples) between the 19th and 20th of June 2019, collaborative networking approaches to innovative services for citizens’ health and sustainability problems related to the Regional Health System were thoroughly discussed.

On the first day of the 2nd International Congress of MeFAVS several topics like “High Technologies in Vascular Surgery” were faced, in line with the general theme of the Forum, and “The diabetic artery disease”, an issue of great interest and relevance, selected as a common research topic among all the countries belonging to the Federation.

The second day was dedicated to the above-mentioned Forum: “Health Technologies: a challenge to be faced in network”.

In this Issue, selected original papers from this conference are reported. All those papers are divided in four panels according to the main topics that were debated:

- Peripheral arterial disease in Diabetics
- Arterial disease
- Venous disease
- Health Technologies and new pathways

### Peripheral arterial disease in Diabetics

Global burden of diabetes, expecting to affect more than 600000 people by 2040, will produce an increase of prevalence of diabetic foot ulcers. Surgical treatment such as debridement and complete revascularization through an “angioplasty first” approach can limit morbidity and mortality in such patients 1.

Diabetic foot teams can guarantee a quick and comprehensive treatment of diabetic foot. A protocol of four steps is proposed, including (1) early diagnosis; (2) urgent surgical treatment of infection; (3) early revascularization within 24 hours; (4) definitive treatment, as wound treatment, reconstruction and orthesis. In such a team, vascular surgeons can play a decisive role to address an effective revascularization leading to lower rates of amputation and mortality 2.

Treating superficial femoral artery lesions in diabetic patients is commonly considered unfeasible because of occlusion risks. In our metanalysis we found that these interventions are not associated with higher reintervention or amputation rates 3.

Diabetic foot osteomyelitis (DFO) is a severe complication of diabetic ischemic, infected foot. When plain X-Ray and MRI, largely used as first- and second-line tests respectively, do not complete diagnosis, 18F-FDG PET/TC and 99mTc-HMPAO-labeled WBC scintigraphy may give definitive information to confirm DFO and potentially proceed to amputation 4.

Endovascular solutions - and Drug Eluting Balloons (DEB) in particular - nowadays have got a central role in the treatment of diabetic arteriopathy, especially in the below-the-knee district, aiming for an accurate, extreme revascularization of the limb 5.

### Arterial disease

There is not a clear consensus on how to treat non-atheromatous carotid lesions. Except for carotid paragangliomas, where surgery offers the best results, endovascular therapies are gaining increasing role in these cases, and aften represent the first-line treatment, such as in radiation-induced sclerosis or fibromuscular dysplasia 6.

Harmonic Focus is an ultrasonic-powered device that using lower temperatures than traditional electrocautery makes the surgery effective and safer for the surrounding structures. HF is multifunctional, capable of sealing, blunt-dissecting, grasping, dividing tissue, producing less smoke than EC, and also reducing operating time, makes surgery safer and more effective than EC. Still there is a paucity of literature about HF in Vascular Surgery in our study we found statistical significant results in terms of incidence of complications of the surgical wound healing comparing HF to EC, founding less complications when HF was used 7.

Splenic artery aneurysms (SAAs) are the most frequent visceral aneurysms. Rupture is the most feared complication, with an incidence ranging from 2.3% to 18% and a mortality rate ranging from 20 to 100%. Percutaneous transcatheter embolization of SAAs with coils is widely accepted as the first line of treatment, because of its safety, its low mortality rate and adequate short- and long-term results. In this report, we present a case of saccular SAA, successfully treated with coil embolization, using the double-microcatheter technique, a further approach to treat SAAs in patients with tortuous vessel’s anatomy 8.

Endovascular atherosclerotic plaques of subclavian artery involving or close to the origin of the vertebral artery expose to the risk of coverage of this vessel after PTA/Stenting. On the other hand, surgery is not recommended due to poor outcomes. DEB may represent a reasonably safe solution to this problem, allowing the use of short stents in case of residual dissection 9.

### Venous disease

Different devices and techniques, like Catheter-Directed Thrombolysis and Percutaneous Mechanical Thrombectomy, now permit an extensive and durable solution again acute deep vein thrombosis in the hands of vascular surgeons 10.

Mechano-chemical ablation (MOCA) is a novel technique for the treatment of saphenous vein insufficiency. Flebogrif, a device causing endothelial damage through radial cutting hooks and chemical ablation with polidocanol foam concentration, is a promising tool for great saphenous vein ablation 11.

### Health Technologies and new pathways

Vascular Surgery profession has been experiencing a true revolution since new drugs, technologies and facilities have been introduced in the latest decades. For example, hybrid rooms and custom-made fenestrated or branched endografts will be the standard of care for thoracic aortic aneurysms. These progressions are, however, time- and cost- consuming and require prepared multidisciplinary teams. It is crucial for vascular surgeons to be well trained to face these big but exciting changes 12.

Colorectal cancer in Campania region shows high prevalence and incidence rates, but the poor technologic equipment often causes migration of patients in those Italian regions which offer more advanced solutions in a shorter time. With the institution of Campania Oncologic Network, it is expected that high-standard treatments will be guaranteed to a larger number of patients through an integrated management system 13. Finally, a novel patented device for automatic processing of clinical data of chronic poly-pathological patients is presented; this paper highlights how new digital technologies can have a huge impact on the traditional healthcare sector^14^.
